# Hemolytic uremic syndrome surveillance to monitor trends in infection with Escherichia coli O157:H7 and other shiga toxin-producing E. coli.

**DOI:** 10.3201/eid0303.970329

**Published:** 1997

**Authors:** B E Mahon, P M Griffin, P S Mead, R V Tauxe


ceftriaxone and convenience of outpatient parenteral therapy, this antibiotic was continued to complete a 10-day course. On extensive questioning, the patient denied contact with any cat, dog, or other animal. He had recently traveled to Nigeria but denied even transient animal contact.

Since 1966, three cases of *P. multocida* epiglottitis have been reported (2-4). Although no direct culture of the epiglottis was performed in the present case, the clinical syndrome and the absence of any other focus accounting for *P. multocida* bacteremia strongly suggest that this organism caused the epiglottitis. Including the present case, three of the four reported cases have occurred since 1993, which suggests that either earlier cases were not recognized or the incidence of this condition may be increasing. In all three previous cases of *P. multocida* epiglottitis, the patients had cats as pets. As in the current case, the clinical features of *P. multocida* epiglottitis were indistinguishable from epiglottitis secondary to more common bacterial pathogens. However, the cases were all associated with positive blood cultures. In contrast, a 23% rate of bacteremia was reported in a series of epiglottitis cases in adults (including patients with blood cultures positive for *Haemophilus influenzae* type b or Group A streptococci)(5).

The vehicle of infection for this patient remains unknown, as human-to-human transmission has not been documented. This case demonstrates that epiglottitis due to *P. multocida*, a rare condition that may be increasing in frequency, need not be accompanied by recognized exposure to animals.

**Michael Glickman and Robert S. Klein**
Montefiore Medical Center and the Albert Einstein College of Medicine, New York, New York, USA

### References

1. Weber DJ, Wolfson JS, Swartz MN, Hooper DC. *Pasteurella multocida* infections: report of 34 cases and review of the literature. Medicine 1984;63:133-54.
2. Johnson RH, Rumans LW. Unusual infections caused by *Pasteurella multocida*. JAMA 1977;237:146-7.
3. Leung R, Jassal J. Pasteurella epiglottitis. Aust N Z J Med 1994;24:218.
4. Rydberg J, White P. *Pasteurella multocida* as a cause of acute epiglottitis. Lancet 1993;341:381.
5. MayoSmith MF, Hirsch PJ, Wodzinski SF, Schiffman FJ. Acute epiglottitis in adults. An eight-year experience in the state of Rhode Island. N Engl J Med 1986;314:1133-9.

### Hemolytic Uremic Syndrome Surveillance to Monitor Trends in Infection with *Escherichia coli* O157:H7 and Other Shiga Toxin-Producing *E. coli*

**To the Editor:** In the past 15 years, knowledge about the role of Shiga toxin-producing *Escherichia coli* (STEC) in human disease has expanded rapidly. The most distinctive complication of STEC infection is diarrhea-associated hemolytic uremic syndrome (HUS), a major cause of acute renal failure in U.S. children. Other manifestations of STEC infection can range from mild diarrhea to severe hemorrhagic colitis, thrombotic thrombocytopenic purpura, and death (1). In the United States, O157 is the most common STEC and causes an estimated 20,000 infections and 250 deaths annually. *E. Coli* O157 outbreaks associated with beef have caused concern among public health workers, clinicians, and the public, prompting major changes in clinical and laboratory practice, meat production, and food preparation. However, critical questions remain unanswered. Have prevention measures decreased risk? Are new sources of STEC infections emerging? Is the incidence of O157 infection changing? How much illness is due to STEC of serotypes other than O157?

Diarrhea-associated HUS is associated with Shiga toxin, which is produced in quantity only by STEC and by *Shigella dysenteriae* type 1; approximately 90% of HUS cases are diarrhea-associated (2,3). In the United States, where *S. dysenteriae* type 1 infections are very rare, STEC infections are the cause of virtually all diarrhea-associated HUS. The incidence of HUS in North America is about three cases per 100,000 children under 5 years of age per year; the rate among older children is somewhat lower, and the rate among adults is not known (2-6). HUS complicates approximately 5% to 10% of O157 infections and an unknown percentage of non-O157 STEC infections (1). Except for supportive care and hemodialysis, no treatment has been shown to decrease the severity of illness or to prevent complications. The sequelae of HUS—death in 3% to 5% of cases (2,3,5) and long-term renal dysfunction in 10% to 30% of survivors (6)—and the lack of specific therapy make prevention critical.

Important changes that may decrease the incidence of STEC infection and HUS in the United States are occurring now. For example, because most outbreaks of STEC infections and HUS have been linked to the consumption of undercooked beef, raw milk, or other products contaminated by the intestinal contents of cattle (1), some U.S. meat producers have changed processing practices to decrease bacterial contamination of meat. As a result of an O157 outbreak caused by consumption of frozen precooked meat patties, federal regulations requiring that this product be cooked adequately to kill O157 were implemented. New requirements that raw meat be labeled with instructions for safe handling and that carcasses be tested for O157 have also recently been implemented nationwide. New vehicles of O157 transmission, for example, dry-cured salami, fermented sausage, and unpasteurized apple juice, have been discovered, prompting the reconsideration of manufacturing processes for these products. Because O157 can be transmitted from person to person (7,8), public health recommendations for control measures to prevent transmission from infected persons have been developed and disseminated (1,8). All these prevention measures show promise; however, their effectiveness has not been documented.

Other changes may not be so salutary. For instance, an increase in international trade in beef and other foods may increase exposure to non-O157 STEC in the United States. Argentina, which has a particularly high incidence of HUS (9), has recently gained approval to start exporting beef to the United States. In Australia, which also exports beef to the United States, a large outbreak of infections with Shiga toxin-producing *E. coli* O111:NM in 1995 was linked to a sausage product; in this outbreak, 23 children became ill with HUS, and one died (10).

Current surveillance methods are unlikely to detect the impact of any of these changes because of two fundamental problems. The first is that changing rates of reported O157 infections and outbreaks do not necessarily reflect actual changes in O157 incidence; it is impossible to tell how much of the marked increase in these reports may be due to greater awareness of rather than actual increase in the incidence of infection. As the public health importance of O157 has become clear, many states have attempted to improve surveillance by mandating reporting of O157 infections. Between 1987 and February 1997, the number of states requiring such reporting increased from 3 to 42 (CDC, unpub. data). In both 1994 and 1995, 32 outbreaks were reported to CDC, the largest numbers ever, bringing the total number of reported U.S. outbreaks to 102; these comprised 2,806 illnesses and 23 deaths (CDC, unpub. data). Both heightened clinician awareness and changes in laboratory stool screening practices (11) have dramatically improved recognition of O157 infections, which has clear public health benefits. For example, if clinical laboratories in Nevada had been screening stool specimens routinely for O157 in 1993, one of the largest clusters of O157 infections ever investigated in the United States might have been recognized and controlled more quickly (12). On the other hand, changing rates of ascertainment of these infections means that O157-based surveillance systems have not been able to show trends in incidence and may not be able to do so reliably in the future.

The second fundamental problem is that surveillance for O157 infections cannot detect trends in non-O157 STEC infections. Non-O157 STEC are not likely to be detected by plating stool specimens on sorbitol-MacConkey agar (13), the method most commonly used to screen for O157. This screening test is based on the fact that, unlike most *E. coli*, very few O157 strains ferment sorbitol rapidly; since most other STEC do ferment sorbitol, this test does not detect them. Yet the non-O157 STEC pose a threat to public health in the United States. Non-O157 STEC, including *E. coli* O111:NM and *E. coli* O104:H21, have caused recent outbreaks detected only because of unusual circumstances. Similarly, sorbitol-positive O157, which recently caused a large outbreak in Germany (pers. comm. Dr. Andrea Ammon, Robert Koch Institute, Germany), would not be detected by current screening practices. In other countries, such as Australia (10) and Argentina (9), non-O157 STEC infections appear to be more common than O157 infections, and in Germany, non-O157 STEC have replaced O157 as the STEC most commonly isolated in HUS cases since 1989 (14). As travel and international trade in food increase, Americans' risk of exposure to these other STEC also increases. However, even major non-O157 STEC outbreaks might not be detected by current U.S. surveillance.

To address these two fundamental problems with current surveillance, the Centers for Disease Control and Prevention has recently initiated active HUS surveillance in sentinel sites, which include Connecticut, Georgia, Minnesota, Oregon, and Alameda County, California and which may be expanded in the future. HUS cases, identified by a definition designed for surveillance (15), in persons under 18 years of age will be prospectively identified through referrals to pediatric nephrologists; these sites have fairly well-defined population bases, so estimating the incidence rate of pediatric HUS will be possible. Including adult HUS may be possible in the future. Because HUS is a distinctive and serious illness, its diagnosis is not likely to be affected by the first surveillance problem, the vagaries of clinical and laboratory practices that can make interpreting O157 isolation data difficult; ascertainment will likely be fairly complete. Therefore, unlike O157-based surveillance, HUS-based surveillance will allow monitoring of trends in the incidence of STEC infection and the examination of the impact of prevention efforts and changing or emerging routes of exposure. HUS cases identified through surveillance are being linked to microbial diagnosis, by culturing patients' stool for O157 and, after screening stool for Shiga toxin-producing colonies, for non-O157 STEC (in the future sera may be tested for O157 or other STEC infections). This linkage will address the second surveillance problem—by differentiating illness caused by the various STEC, linked microbial diagnosis will allow detection of trends in the incidence of non-O157 STEC as well as O157. Because passive surveillance for HUS, which has been conducted by many states and continues on a national level, lacks the microbial diagnostic component of the active surveillance system, it cannot show these trends. Active surveillance for HUS will be an efficient approach to STEC surveillance because essentially all patients with diarrhea-associated HUS have STEC infections—HUS is a more potent indicator than any other clinical syndrome.

This surveillance effort can provide the framework for future investigations in several areas. Clinical and immunologic risk factors for HUS following O157 infection could be defined through case-control studies using as controls patients with O157 infection but without HUS. Risk factors for infection with non-O157 STEC could be characterized. Serum collected from patients with non-O157 STEC infections could be used to develop serologic tests for infection with these organisms. Methods for Shiga toxin identification in stool could be evaluated. Finally, HUS treatments could be evaluated in a well-defined patient population and study network.

Reliable surveillance data are critical to targeting prevention efforts and defining their success. A national HUS surveillance system will provide the information needed to measure the impact of new and changing vehicles of STEC transmission, evaluate the effectiveness of prevention measures, and detect illness caused by non-O157 STEC.

**Barbara E. Mahon,* Patricia M. Griffin,† Paul S. Mead,† Robert V. Tauxe†**
*UMDNJ-Robert Wood Johnson Medical School, New Brunswick, New Jersey, USA;
†Centers for Disease Control and Prevention, Atlanta, Georgia, USA

### References

1. Griffin PM. *Escherichia coli* O157:H7 and other enterohemorrhagic *Escherichia coli*. In: Blaser MJ, Smith PD, Ravdin JI, Greenberg HB, Guerrant RL, editors. Infections of the gastrointestinal tract. New York: Raven Press, Ltd., 1995. p. 739-61.
2. Martin D, MacDonald K, White K, Soler J, Osterholm M. The epidemiology and clinical aspects of the hemolytic uremic syndrome in Minnesota. N Engl J Med 1990;323:1161-7.
3. Rowe PC, Orrbine E, Wells GA, McLaine PN, Members of the Canadian Pediatric Kidney Disease Reference Center. Epidemiology of hemolytic-uremic syndrome in Canadian children from 1986 to 1988. J Pediatr 1991;119:218-24.
4. Kinney J, Gross T, Porter C, Rogers M, Schonberger L, Hurwitz E. Hemolytic-uremic syndrome: a population-based study in Washington, DC and Baltimore, Maryland. Am J Public Health 1988;78:64-5.
5. Tarr PI, Hickman RO. Hemolytic uremic syndrome epidemiology: a population-based study in King County, Washington, 1971 to 1980. Pediatrics 1987;80:41-5.
6. Siegler R, Pavia A, Christofferson R, Milligan M. A 20-year population-based study of postdiarrheal hemolytic uremic syndrome in Utah. Pediatrics 1994;96:35-40.
7. Rowe PC, Orrbine E, Ogborn M, Wells GA, Winther W, Lior H, McLaine PN. Epidemic *Escherichia coli* O157:H7 gastroenteritis and hemolytic-uremic syndrome in a Canadian Inuit community: intestinal illness in family members as a risk factor. J Pediatrics 1994;124:21-6.
8. Belongia E, Osterholm M, Soler J, Ammend D, Braun J, MacDonald K. Transmission of *Escherichia coli* O157:H7 infection in Minnesota child day-care facilities. JAMA 1993;269:883-8.
9. Lopez EL, Diaz M, Grinstein S, Devoto S, Mendilaharzu F, Murray BE, et al. Hemolytic uremic syndrome and diarrhea in Argentine children: the role of Shiga-like toxins. J Infect Dis 1989;160:469-75.
10. Goldwater PN, Bettelheim KA. An outbreak of hemolytic uremic syndrome due to *Escherichia coli* O157:H7: or was it? Emerg Infect Dis 1996;2:153-4.

11. Boyce TG, Pemberton AG, Wells JG, Griffin PM. Screening for *Escherichia coli* O157:H7—a nationwide survey of clinical laboratories. J Clin Microbiol 1995;33:3275-7.
12. Cieslak PR, Noble SJ, Maxson DJ, Empey LC, Ravenholt O, Legarza G, et al. Hamburger-associated *Escherichia coli* O157:H7 infection in Las Vegas: a hidden epidemic. Am J Public Health 1997;87:176-80.
13. March SB, Ratnam S. Sorbitol-MacConkey medium for detection of *Escherichia coli* O157 associated with hemorrhagic colitis. J Clin Microbiol 1986;23:869-72.
14. Huppertz H, Busch D, Schmidt H, Aleksic S, Karch H. Diarrhea in young children associated with *Escherichia coli* non-O157 organisms that produce Shiga-like toxin. J Pediatr 1996;128:341-6.
15. Centers for Disease Control and Prevention. Case definitions for infectious conditions under public health surveillance. MMWR Morb Mortal Wkly Rep 1997;46:17.
